# Multivariate data analysis: Validation of an instrument for the evaluation of teaching digital competence

**DOI:** 10.12688/f1000research.135194.2

**Published:** 2023-11-06

**Authors:** Andrés Santiago Cisneros-Barahona, Luis Marqués-Molías, Nicolay Samaniego-Erazo, Catalina Mejía-Granizo, Gabriela De la Cruz-Fernández

**Affiliations:** 1 Universitat Rovira i Virgili, Tarragona, Catalonia, 43007, Spain; 2 Universidad Nacional de Chimborazo, Riobamba, Chimborazo Province, 060150, Ecuador; 3 Banco Guayaquil, Riobamba, Chimborazo, 060150, Ecuador

**Keywords:** technological literacy, teacher training, educational research, university teachers, confirmatory factor analysis

## Abstract

**Background:** Technology plays a fundamental role to achieve higher education key learning objectives. Digital competence (DC) is defined as a set of skills, knowledge, abilities, and attitudes in technological aspects. It is necessary to employ an effective training action plan in higher education institutions to advance towards a level of teaching digital competence (TDC). The objective of this study was to validate the COMDID A instrument to assess Teaching Digital Competence (TDC) of active teachers, through a confirmatory factor and internal reliability analysis.

**Methods:** The research was developed within a descriptive-correlational scope and a non-experimental-cross-sectional design to validate the dimensionality and reliability of the COMDID A instrument and evaluate the self-perceived digital competence of active teachers. The population was made up of 690 professors who were part of the teaching staff of the National University of Chimborazo, Ecuador, in the first academic period of the year 2021. The sample was probabilistic, in a simple random scheme, the percentage of potential error admitted was 3%. The representativeness of the sample was 50%, and the confidence level was 97%. A total of 511 teachers completed the questionnaire compared to the 452 individuals needed.

**Results:** The instrument was robust, and it was reliable for the calculated sample. There were correlations between the variables, and the statistical calculation ensured the development of the multivariate analysis to validate the dimensionality of the instrument. Moreover, the correct dimensionality was determined through a confirmatory analysis and high reliability of the instrument.

**Conclusions:** The calculated factorial scores were defined in order for further studies to be carried out. It is important to apply confirmatory factor analysis in educational technology research to validate the dimensionality of data collection instruments.

## Introduction

In 21st century society, which is digitally rich, the construction of a comprehensive and inclusive higher education is essential, one which attends to various actors’ needs and linking the university with society ( Domingo-Coscollola *et al*., 2020). To accomplish this task, it is necessary for academic personnel to reach at least a medium DC level so universities must invest time and resources in the training of their professors ( Amaya Amaya *et al*., 2018; Cisneros-Barahona, Marqués-Molías, *et al*., 2022b; Reyes, Cárdenas, 2018; Sánchez-Caballé *et al*., 2020).

Nowadays, there is a gap between teacher's skills and the deficient academic training they receive to achieve them. This is due to the confusion about the conceptualization of digital competences and then to the limitations of developing efficient digital training plans ( Biel & Ramos, 2019; Malagón Terrón & Graell Martín, 2022), through public policies that strengthen the inclusion and treatment of these capacities in initial and continuous teachers’ training ( Cabero-Almenara *et al*., 2021; Garita-González *et al*., 2019; Guillén-Gámez *et al*., 2021; Silva *et al*., 2019).

The importance of information and communication technologies (ICTs) in higher education lies in the improvement of teaching and learning processes through the inclusion of DCs, which also improve students’ training and professional performance ( Fernández-Márquez *et al*., 2018; García-Ruiz *et al*., 2023; Juárez Arall & Marqués Molías, 2019).

DC is defined as the integration of knowledge, skills, attitudes, capacities ( Rangel, 2015; Vivar, 2014; Zepeda *et al*., 2019), whose purpose is the use of digital technologies in a responsible, safe, and critical manner ( Ferrari, 2013).

It is essential to understand the distinction between DC and TDC. Internationally, the term “digital literacy” is used to refer to DC, while in the European context, the concept of DC is used equivalently ( Almås & Krumsvik, 2007). DC is defined as a comprehensive set of values, beliefs, knowledge, skills, and attitudes in the technological, informational, multimedia, and communicative fields that blend into a multiple and complex competence ( Gisbert Cervera & Esteve Mon, 2011). Its fundamental purpose lies in the effective management of information for knowledge construction ( Gutiérrez, 2011). This entails secure, critical, and responsible use of ICT for educational, professional, and social purposes, as well as interaction with them.

On the other hand, the concept of TDC refers to a complex professional competence that encompasses a set of knowledge, skills, and attitudes that educators must possess and simultaneously apply to use DT in their pedagogical practice ( Lázaro-Cantabrana *et al*., 2019). It is also defined as the competences that 21st-century teachers must cultivate to enhance their educational practice and promote their continuous professional development ( INTEF, 2017).

Systematic literature reviews have been carried out, supported by meta-analysis and bibliometrics, to explain the concept of teaching digital competence (TDC) and to categorize theoretical aspects that make it possible to interpret the evaluation effects easily and improve these skills ( Cisneros-Barahona, Marqués-Molías, *et al*., 2022a; Cisneros-Barahona, Marqués Molías, *et al*., 2023a; Cisneros-Barahona, Marqués-Molías, Samaniego-Erazo, Uvidia-Fassler, & De la Cruz-Fernández, 2023d; Cisneros-Barahona, Marqués-Molías, Samaniego-Erazo, Uvidia-Fassler, De la Cruz-Fernández, *et al*., 2023; Delfín & Pirela, 2017; Gisbert Cervera *et al*., 2016; Marqués-Molías *et al*., 2016; Verdú-Pina *et al*., 2022).

In this regard, models with dimensions, standards, and indicators have been designed to evaluate DC levels from various perspectives using various instruments ( Almås & Krumsvik, 2007; Beetham *et al*., 2009; Butcher, 2019; Caena & Redecker, 2019; Campo *et al*., 2013; CDEST, 2002; Elliot *et al*., 2011; INTEF, 2017; ISTE, 2000, 2008; Lázaro-Cantabrana *et al*., 2019; Palau *et al*., 2019; Redecker, 2017; Trilling, 2002), that have enabled, on the one side, the appreciation of the problems related to the deficiency of the formative aspects in teachers, as part of the strategies implemented to reach adequate levels of TDC ( Angulo *et al*., 2015; Cisneros-Barahona, Marques-Molías, *et al*., 2022; Fernández Cruz & Fernández Díaz, 2016; Fernández-Diaz *et al*., 2021; Gutiérrez & Cabero-Almenara, 2016; Morales Capilla *et al*., 2014; Ramírez García & González Fernández, 2016; Silva Quiroz & Miranda Arredondo, 2020); and, on the other side, to generate more competent professionals ( Juárez Arall & Marqués Molías, 2019).

According to the relationships that the TDC has with other variables, there are studies that point out the importance of age ( Cabero-Almenara *et al*., 2020; Usart Rodríguez *et al*., 2020), generation ( Basantes-Andrade *et al*., 2020) or gender ( De la Iglesia *et al*., 2020; Zhao *et al*., 2021). In contrast, there are studies in which the variables of gender ( Guillén-Gámez *et al*., 2021), age, and type of degree are considered inconclusive, and the relevance is assigned to the variable of the teachers’ attitude toward the use of technological tools ( Galindo-Domínguez & Bezanilla, 2021).

On the other hand, the evaluation instruments must be reliable and valid to generalize their use in any context ( Hernández Sampieri *et al*., 2014b; Larraz, 2013). Reliability is the degree to which repeated application of an instrument produces the same results, while validity is the degree to which an instrument measures a variable for real.

The COMDID A self-perception instrument ( Lázaro & Gisbert, 2015; Lázaro-Cantabrana *et al*., 2018), is a self-perception rubric that characterizes the teacher, relating him to the level of TDC. The instrument has four dimensions: 1. Didactic, curricular, and methodological (six indicators); 2. Planning, organization and management of spaces and digital technological resources (five indicators); 3. Relational, ethical and security (five indicators), and 4. Personal and professional (six indicators) and proposes five response options related to a rating scale for each of the 22 indicators (0, 25, 50, 75 and 100).

The COMDID A instrument has been through several design and development stages ( Usart Rodríguez *et al*., 2020): 1. Literature review on which the instrument is based; 2. Items design that are part of the questionnaire; 3. Validation by experts and, 4. Validation of factorial structure and internal reliability in relation to age, gender and access to the university in the version for initial teacher’s training ( Lázaro & Gisbert, 2015; Lázaro-Cantabrana *et al*., 2018).

Cronbach's alpha is an internal consistency measure and makes it possible to quantify the correlation that exists between the items that compound a scale ( Cervantes, 2005; Cronbach, 1951; González & Pazmiño, 2015).

Factorial analysis is a multivariate statistical technique, which seeks to obtain a reduced set of unobserved or abstract variables (common factors), which reproduce or represent the correlation shared by the observed variables. In other words, it makes it possible to facilitate the interpretation of a group of observed variables by reducing their number to a few that represent the common causes shared by the original variables, without losing the information ( Mateos-Aparicio & Hernández Estrada, 2021).

The primary purpose of this research is to validate the COMDID A tool in order to assess the DC of active teachers, using Confirmatory Factor Analysis and evaluating its internal reliability. Furthermore, it seeks to expand knowledge in the measurement of teacher digital competence, thereby contributing to the advancement of research in this field.

## Methods

### Ethical considerations

Ethical approval was obtained on December 23rd, 2021 from the Society and Environment Ethic Research Committee (CEIPSA (in Spanish)), Universitat Rovira i Virgili, CEIPSA-2021-PR-0035. All participants were asked to sign a written informed consent before enrolment.

### Instrument

The instrument employed in this study is the COMDID A questionnaire, designed for the purpose of assessing TDC currently in active practice. The selection of this instrument is grounded in its notable strengths, which have been highlighted in previous validation processes ( Lázaro & Gisbert, 2015; Usart Rodríguez *et al.*, 2020). Furthermore, it is imperative to underline that this questionnaire has been adapted to the Latin American context, rendering it particularly pertinent to our research objectives ( Lázaro-Cantabrana *et al.*, 2018).

**Figure 1.**
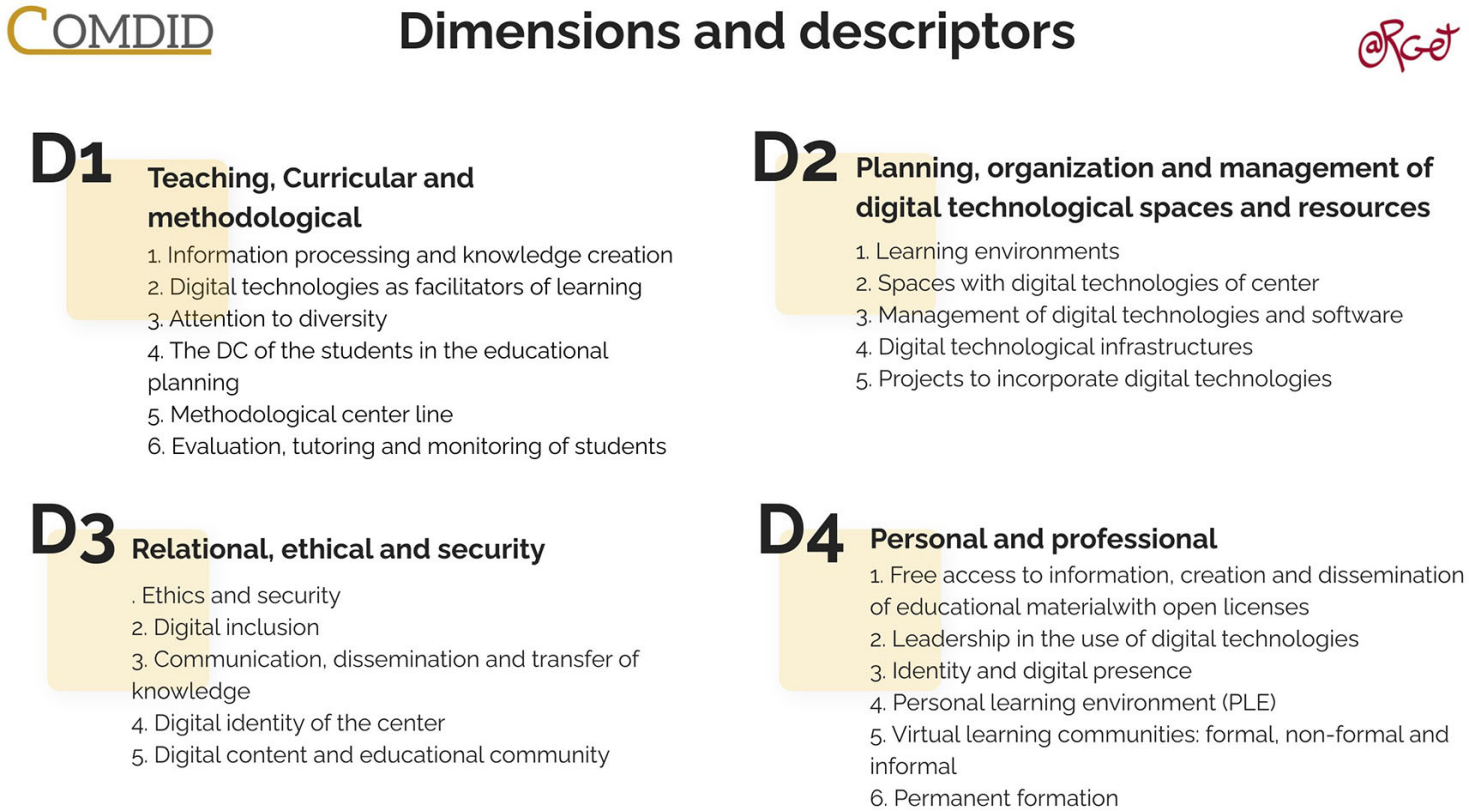
Dimensions and Descriptors of Digital Competence in the COMDID Model. Source: ARGET Research Group, Universitat Rovira i Virgili.

The instrument comprises four distinct dimensions, each with its respective indicators. Firstly, we encounter the dimension of Teaching, Curricular, and Methodological, encompassing 6 indicators. The second dimension addresses the Planning, Organization, and Management of Digital Technological Spaces and Resources, comprising a total of 5 indicators. The third dimension focuses on Relational, Ethical, and Security aspects, composed of 5 indicators. Lastly, the fourth dimension, termed Personal and Professional, encompasses 6 indicators. These details are depicted in Figure 1.

A scale from 0 to 100 defined the level of development of TDC in each dimension, with intervals: 1. Not started (N0), 2. Beginner (N1), 3. Medium (N2), 4. Expert (N3) and 5. Transformer (N4).

The questionnaire employs a rating scale consisting of five response options, corresponding to a scoring scale covering values (0, 25, 50, 75, and 100). This scale facilitates the assessment of each indicator based on its level of development. To categorize the level of Digital Competence in both individual dimensions and globally, a numerical scale ranging from 0 to 100 has been established. This scale is defined in intervals as follows:

• Level 0 (L0) (0-12.4).
• Level 1 (L1) (12.5-37.4).
• Level 2 (L2) (37.5-62.4).
• Level 3 (L3) (62.5-87.4).
• Level 4 (L4) (87.5-100).

The study aims to validate the COMDID A instrument to assess active teachers’ DC through a confirmatory factor analysis and internal reliability.

The scope was descriptive-correlational, and the design was a cross-sectional non-experimental ( Arias, 1999, 2012; Arnal *et al*., 1992; Bisquerra, 1989; Bisquerra *et al*., 2009; Hernández Sampieri *et al*., 2014b; Ramos-Galarza, 2020).

Equation 1 was applied to calculate the sample size to estimate the portion of the desired population with a known confidence interval ( Badii *et al*., 2008):

$$n=\frac{Nz^{2}\;\mathrm{pq}}{E^{2}\left(N-1\right)+z^{2}\;\mathrm{pq}}$$ (1)

Where:

*n*: required minimum sample size.

*N*: Population size.

*z*: Z statistic for a level of confidence.

*p*: Expected proportion.

*q*: Expected proportion.

*E*: margin of error.

The population for this study was made up of 690 professors who were part of the teaching staff of the National University of Chimborazo, Ecuador, during the second academic period of 2021. The sample is probabilistic in a simple random scheme ( Hernández Sampieri *et al*., 2014a; Kerlinger & Lee, 1985), the admitted potential error rate was 3%. The representativeness of the sample was 50%, and the confidence level was 97%. A total of 511 teachers completed the questionnaire, compared to the 452 individuals needed, according to the sample requirement ( Badii *et al*., 2008) and, above the five samples per item required to confirm structures (110 samples according to the 22 items) ( Hair *et al*., 2010).

The reliability of the instrument was calculated through Cronbach's Alpha ( Cronbach, 1951) using IBM SPSS Statistical Software, version 28.0.1.1(15). The dimensional constructs of COMDID A for active teachers were validated through confirmatory factor analysis, which also identified the latent factors that simplified the relationships established in the set of observed variables ( López-Aguado & Gutiérrez-Provecho, 2019).

The intention was to confirm the structure of four factors that were related to the construction and theoretical validation of the instrument, through the principal component extraction method, with Kaiser-Meyer-Olkin (KMO) measure and Bartlett's test of sphericity; on a set of 22 indicators or items to try to reduce the amount of data observed and, thus, identify the four theoretical dimensions. A Varimax rotation was also used since they were orthogonal factors. The sample was 511 individuals, above the five samples per item required for this type of analysis (110 samples) ( Hair *et al*., 2010).

### Limitations

This study comes with limitations that should be taken into consideration. It is essential to highlight that it relied on a self-perception questionnaire, meaning that participants' responses were based on their own subjective perception. This may not accurately reflect the true level of CDD. Therefore, it is suggested that future research utilize correlational analyses to enable a more in-depth exploration of how variables impact CDD and how different components of this competency are interrelated.

## Results

### Reliability

The Cronbach coefficient was used to validate the instrument’s reliability as a statistic to estimate the reliability of any compound obtained from the sum of several measurements ( Cronbach, 1951).

This validation was used as an analysis technique, in the second period of 2021, with the sample of 511 teachers. Results can be seen in Table 1, according to the dimensions of the instrument:

• Dimension 1 (D1): Teaching, Curricular and methodological
• Dimension 2 (D2): Planning, organization and management of digital technological spaces and resources
• Dimension 3 (D3): Relational, ethical and security
• Dimension 4 (D4): Personal and professional

**Table 1.** Statistics of reliability of the indicators of the dimensions of the COMDID A instrument.

Overall dimensions			
Cronbach’s Alpha		No. of items	
0.956		22	
**Dimension 1**		**Dimension 2**	
Cronbach’s Alpha	No. of items	Cronbach’s Alpha	No. of items
0.836	6	0.871	5
**Dimension 3**		**Dimension 4**	
Cronbach’s Alpha	No. of items	Cronbach’s Alpha	No. of items
0.857	5	0.891	6

### Validity: Confirmatory factor analysis

The stages of analysis are ( Mateos-Aparicio & Hernández Estrada, 2021):

• Stage 1. Prior assumptions of the analysis.
• Stage 2. Extraction of factors.
• Stage 3. Rotation of factors.
• Stage 4. Determination of factorial scores.

*Stage 1. Prior assumptions of the analysis*

**Figure 2.**
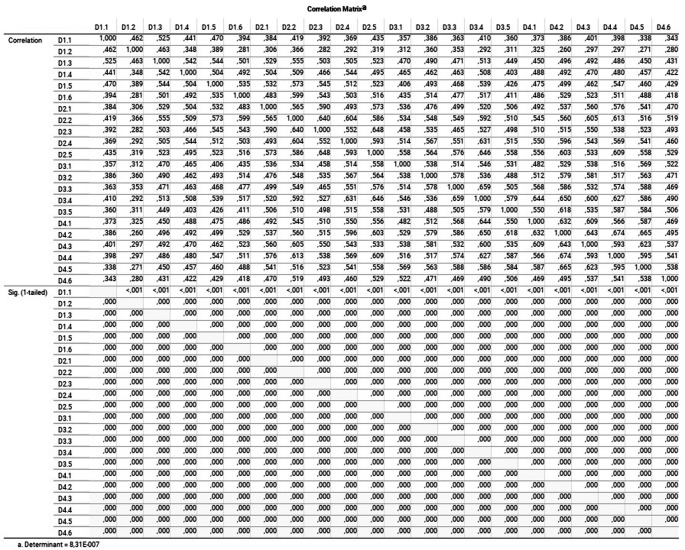
Correlation matrix and determinant: linearity and correlation coefficients of each variable.

Table 2 shows the Kaiser-Meyer-Olkin (KMO) measure with a sampling adequacy of 0.974. Bartlett's test of sphericity presents the statistic value (7025.987), because of the low value of significance (0). Figure 2 shows the correlation matrix and its determinant close to 0 ( $8.310\;\times \;10^{-7})$ ( Cisneros-Barahona *et al.*, 2023b).

**Table 2.** Kaiser-Meyer-Olkin (KMO) and Bartlett's Test - A measure of sampling adequacy.

**Kaiser-Meyer-Olkin Measure of Sampling Adequacy.**		0.974
**Bartlett's Test of Sphericity**	Approx. Chi-Square	7025.987
	df	231
	Sig.	0.000

*Stage 2. Extraction of factors*

**Figure 3.**
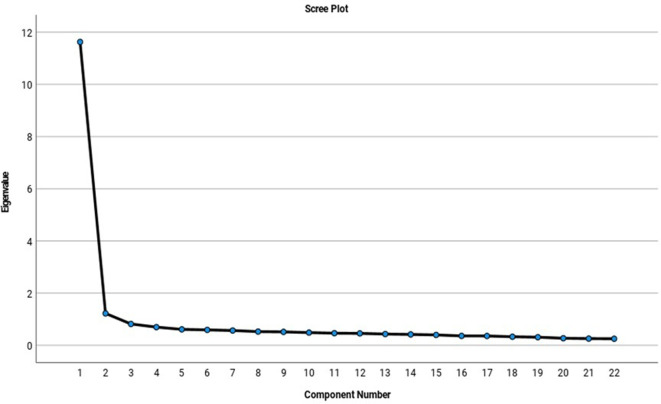
Scree plot: fall contrast criteria.

**Figure 4.**
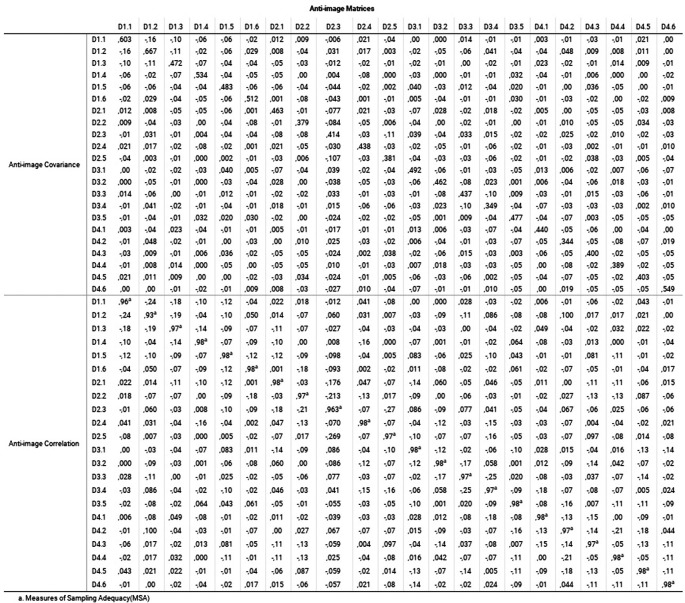
Anti-image matrices: Measure of the sampling adequacy (MSA) index.

The confirmatory factor analysis through the principal component extraction method and with the extraction criterion of a fixed value of 4 explained the variance value of 65.31%, see Table 3. Figure 3 shows the scree plot that indicates that four factors were viable according to the fall contrast criterion. Figure 4 reveals the Measure of Sampling Adequacy (MSA) index, through the values of the main diagonal of the anti-image matrices.

**Table 3.** Total variance explained.

Component	Initial eigenvalues			Extraction sums of squared loadings			Rotation sums of squared loadings		
	Total	% of Variance	Cumulative %	Total	% of Variance	Cumulative %	Total	% of Variance	Cumulative %
**1**	11.627	52.851	52.851	11.627	52.851	**52.851**	5.012	22.781	22.781
**2**	1.224	5.564	58.415	1.224	5.564	**58.415**	3.969	18.043	40.824
**3**	0.819	3.725	62.140	.819	3.725	**62.140**	3.181	14.461	55.285
**4**	.699	3.176	65.316	.699	3.176	**65.316**	2.207	10.031	**65.316**
**5**	.613	2.785	68.101						
**6**	.592	2.693	70.793						
**7**	.569	2.584	73.378						
**8**	.527	2.395	75.772						
**9**	.516	2.346	78.118						
**10**	.489	2.224	80.342						
**11**	.466	2.118	82.460						
**12**	.458	2.084	84.544						
**13**	.433	1.968	86.512						
**14**	.419	1.903	88.415						
**15**	.400	1.816	90.231						
**16**	.362	1.645	91.876						
**17**	.360	1.636	93.512						
**18**	.330	1.499	95.012						
**19**	.313	1.422	96.434						
**20**	.273	1.239	97.673						
**21**	.259	1.176	98.849						
**22**	.253	1.151	100.000						

Extraction Method: Principal Component Analysis.

*Stage 3. Rotation of factors*

An orthogonal rotation is applied, using the Varimax method. In Table 3, it can be seen how the value of the total variance explained is the same for the non-rotated matrix and for the rotated matrix (65.316), even though the accumulated variances of each factor do not hold.

*Stage 4. Determination of factorial scores*

The communalities coefficients are shown in Table 4. Table 5 notes the score obtained in each of the cases of the extracted components to estimate factors.

**Table 4.** Communalities.

	Initial	Extraction
**D1.1**	1.000	.630
**D1.2**	1.000	.784
**D1.3**	1.000	.647
**D1.4**	1.000	.550
**D1.5**	1.000	.641
**D1.6**	1.000	.637
**D2.1**	1.000	.640
**D2.2**	1.000	.674
**D2.3**	1.000	.715
**D2.4**	1.000	.631
**D2.5**	1.000	.636
**D3.1**	1.000	.627
**D3.2**	1.000	.555
**D3.3**	1.000	.661
**D3.4**	1.000	.722
**D3.5**	1.000	.635
**D4.1**	1.000	.658
**D4.2**	1.000	.719
**D4.3**	1.000	.622
**D4.4**	1.000	.637
**D4.5**	1.000	.677
**D4.6**	1.000	.671

Extraction Method: Principal Component Analysis.

**Table 5.** Coefficient Matrix of Component Score.

	Component			
	1	2	3	4
D1.1	-.102	.003	-.071	.450
D1.2	-.047	-.276	.001	.675
D1.3	-.134	.134	-.023	.257
D1.4	.025	.207	-.209	.095
D1.5	-.093	.335	-.169	.057
D1.6	-.012	.399	-.225	-.126
D2.1	-.253	.183	.306	-.072
D2.2	-.105	.258	.032	-.065
D2.3	-.267	.366	.192	-.178
D2.4	.196	.133	-.215	-.082
D2.5	-.030	.096	.112	-.058
D3.1	-.075	-.192	.409	.055
D3.2	.126	-.029	-.028	.045
D3.3	.351	-.159	-.189	.065
D3.4	.320	-.031	-.214	-.038
D3.5	.089	-.259	.285	.035
D4.1	.342	-.109	-.189	.002
D4.2	.288	-.085	-.078	-.084
D4.3	.080	-.040	.119	-.055
D4.4	.083	.012	.073	-.078
D4.5	.164	-.177	.170	-.065
D4.6	-.241	-.115	.579	-.011

Extraction Method: Principal Component Analysis.

Rotation method: Varimax with Kaiser normalization.

*Principal component analysis:* with one Varimax rotation for one extraction of four principal components, the rotation has converged in six iterations, and an explained variance greater than 65.316% was obtained ( Table 3). Table 6 states the rotated components ordered according to the instrument factors. Values less than 0.3 are excluded for samples superior to 350 people by using SPSS ( Hair *et al*., 2010).

**Table 6.** Rotated component matrix ^a^.

	Component			
	D3	D2	D4	D1
**D1.1**				.683
**D1.2**				.858
**D1.3**				.525
**D1.4**				.341
**D1.5**				.332
**D1.6**		.683		
**D2.1**		.520		
**D2.2**		.611		
**D2.3**		.673		
**D2.4**		.480		
**D2.5**		.469		
**D3.1**	.392			
**D3.2**	.526			
**D3.3**	.708			
**D3.4**	.718			
**D3.5**	.529			
**D4.1**	.709			
**D4.2**			.329	
**D4.3**			.445	
**D4.4**			.417	
**D4.5**			.493	
**D4.6**			.723	

Extraction method: Principal component analysis.

Rotation method: Varimax with Kaiser normalization.

^a^ Rotation converged in six iterations.

## Discussion

Internal consistency reliability is a way to estimate the equivalence of the components among themselves, and it indicates the inner correlation between the variables of the instrument by separating the variation of the common factors and the variation of the unique factors of each item. In this sense, the reliability of the instrument was evaluated through the calculation of Cronbach's Alpha coefficient for the complete instrument, understanding an alpha calculation for each of the dimensions ( Campo-Arias, 2006; Ledesma *et al*., 2002; Merino Soto & Lautenschlager, 2003; Torres, 2021), which gave the following results: For Dimension 1. Didactic, curricular, and methodological (α=0.836); for Dimension 2. Planning, organization and management of spaces and digital technological resources (α= 0.871); for Dimension 3. Relational, ethics and security (α=0.857), and Dimension 4. Personal and professional (α=0.891). The α of the complete instrument was 0.956, data that allows us to confirm that the instrument has high internal reliability.

When observing the correlation matrix of the indicators, it was difficult to define for certain the number of correlation coefficients greater than 0.5 ( Mateos-Aparicio & Hernández Estrada, 2021); because of that the determinant of the correlation matrix was used. If this value is closer to 0, it will imply a more significant association of the variables with each other, reaching the total dependence if it is 0 (all the elements of the matrix to 1). In the study, it was necessary to calculate the determinant since not all the values of the matrix were 1 (determinant = 0, total dependency), nor the values of the main diagonal at 1 and the rest at 0 (identity matrix) (determinant = 1, total independence). In our case, we have the relation to 1 on the diagonal of the correlation matrix for each variable with itself, and outside of this diagonal, the correlation coefficients of each pair of variables, with a calculated determinant of 8.310 x 10 ^-7^. At first glance, the determinant is quite close to 0. However, considering that the information comes from a sample and, to define an adequate degree of correlation between the variables, the Bartlett test of sphericity was calculated.

Bartlett's sphericity test proves the null hypothesis that the variables analyzed are not correlated in the sample, which means that it contrasts with the hypothesis that the correlation matrix is the identity matrix (the intercorrelations between the variables are zero, except for the main diagonal, which is 1). If this were true, there is no correlation between variables, and it would not make sense to do a factor analysis. Visually, the null hypothesis is rejected, since the correlation matrix is not the identity matrix, in fact, it is significantly different, which implies that there are high values of association. However, if the null hypothesis is not rejected for a level of significance, the variables would not be sufficiently correlated, and it would not make any sense to do a factor analysis. The high value of the statistic (7025.987) indicates that it belongs to the critical region, data that is confirmed with the low value of significance (0); these values allow the rejection of the null hypothesis. However, having a sample size greater than 100, the null hypothesis is always rejected since the sample size is predominant when calculating the statistic. To solve this problem, we chose the KMO measure, which compares the observed correlation coefficients with the partial correlation coefficients for all variables ( Garmendía, 2007; Mateos-Aparicio & Hernández Estrada, 2021).

The structure of the instrument fitted the sample through hypothesis contrasts. The KMO measure showed a sampling adequacy of 0.974 that allowed us to be sure that the sample data were appropriate to perform a factor analysis (if it was higher at 0.90, it would be considered excellent sample adequacy of factorial data matrices (Kaiser, 1970)), between 0.8 and 0.9 means that the analysis is good or very good ( Mateos-Aparicio & Hernández Estrada, 2021). Additionally, the value of the determinant of the correlation matrix was close to 0, which allowed us to confirm that the intercorrelation degree of the variables was quite high.

When inspecting the sedimentation graph ( Figure 2), it was observed that four factors (dimensions) were viable according to the falling contrast criterion since the inflection point was located where the eigenvalues stop forming a slope and begin to generate a low inclination fall from the fifth factor ( Cattell, 1966; Hair JR *et al*., 2010; Pérez & Medrano, 2010).

In the anti-image matrix of Figure 3, the values of the complete matrix indicate the coefficients of partial relationships and explain the correlations not explained by the common factors. MSA is based on KMO; therefore, the interpretation of MSA in the main diagonal is like the coefficient. In this case, all the values were greater than 0.9, so the elimination of any variable was not considered, in addition to the fact that the elements outside the diagonal were less than 0.5 ( Mateos-Aparicio & Hernández Estrada, 2021). It implies that the application of factor analysis was adequate in this sample ( Garmendía, 2007).

The initial communalities in Table 4 measure the percentage of variance in a variable explained by all the factors together, and it can be interpreted as the reliability of the indicator ( Garmendía, 2007). They appear in 1 because, in the principal component analysis (PCA), as many factors are calculated as original variables; this means that the total variance of the original variables is reproduced. The communalities are also observed after the extraction; the greater the communality, the better the variables will be represented by the factorial model. In this case, all the communalities were greater than 0.5, which means that they reproduced more than half of their variance, data that indicate that our variables were very well represented ( Mateos-Aparicio & Hernández Estrada, 2021).

A confirmatory factor analysis was developed, with the principal component extraction method, as it is the most appropriate method to estimate the factorial model and because of having the advantage of always providing a solution ( López-Aguado & Gutiérrez-Provecho, 2019) as the factors explain the total variance correctly. The extraction criterion was a fixed value of 4 at the rate of each one of the dimensions of the questionnaire and through a Varimax rotation (it is the best known and applied method ( Mateos-Aparicio & Hernández Estrada, 2021)) to minimize the number of variables with high load in each factor and to simplify the interpretation of the factors. This means that it simplifies the components to have high correlations with few variables and it is one of the properties of the Varimax method since the total variance explained before and after rotating is maintained, but not the total variance of each factor.

An explained variance effect of 65.31% was obtained to see the original structure of the instrument in the sample, with four factors in Table 3 (with a reduction of dimensionality from 22 to 4). This implies that there are enough factors (greater than 60%) ( Hair *et al*., 2010) to determine that the factorial structure is correct for the sample, rediscovering the four theoretical dimensions. Thus, the importance of the application of confirmatory factor analysis in educational technology research is determined to validate the dimensionality of the data collection instruments.

The confirmatory factorial analysis determines that the factorial structure is correct for the sample, rediscovering the four theoretical dimensions (1. Didactics, Curricular and methodological; 2. Planning, organization, and management of spaces and digital technological resources; 3. Relational, ethics and security, and 4. Personal and professional ( Lázaro *et al*., 2018; Lázaro & Gisbert, 2015).

It was observed that not all the items had the necessary weights to be located univocally in a factor; this is due to the high association that the dimensions have concerning the formative aspects of teachers, the organization and management of resources, and strategic area ( Usart Rodríguez *et al*., 2020).

Factorial scores were calculated ( Table 5) for each case and in each of the extracted components to estimate factors, to carry out subsequent studies, and to replace the set of original variables with the set of principal components that represent them (reduced). In addition, it was observed that the instrument was robust for evaluating the DC of active teachers.

## Conclusions

The study has yielded significant findings regarding the reliability and validity of the COMDID A instrument. In the initial phase, a reliability analysis of the questionnaire was conducted using Cronbach's Alpha coefficient to assess the entire set of questions. The results obtained solidly confirm that the instrument is highly reliable for the sampled population.

The Confirmatory Factor Analysis (CFA) performed in this study has enhanced the accuracy and validity of the constructs measured by COMDID A, adding quality and credibility to research results that utilize this instrument. Beyond validation, this analysis has provided the valuable opportunity to refine the model by identifying any potential theoretical issues requiring revision. This process has focused on improving measurement accuracy and minimizing potential margin of error.

It is noteworthy that the proposed construct structure in the COMDID A measurement instrument has been confirmed to be congruent with the data collected from the sample. This finding implies that the designed items are genuinely related to the theoretical model upon which the instrument is based, making it a suitable tool for explaining the relationship between observed variables.

Additionally, a dimensional structure of underlying factors in the dataset has been robustly established. Items have been grouped according to previously defined dimensions, showing a high correlation among them. It has been verified that the four dimensions are valid and measure distinct concepts. Therefore, we can confidently assert that the instrument exhibits high internal consistency in measurements, significantly contributing to its reliability.

The correlation matrix allows us to observe how different dimensions (D1, D2, D3, D4) are interrelated and how each individual variable relates to the others. This is crucial for understanding the underlying relationships between variables in the COMDID A model. Convergent validity is evident as variables within the same dimension tend to correlate more strongly with each other than with variables from other dimensions. This supports the notion that variables within a dimension are related and measure the same underlying construct.

Regarding discriminant validity, correlations between variables from different dimensions are generally lower than correlations within the same dimension, indicating that the dimensions are distinct from each other. The determinant value is relevant and allows verification of multicollinearity among variables, which is essential for interpreting CFA results.

The high value of the Kaiser-Meyer-Olkin (KMO) index at 0.974 indicates that the data are suitable for conducting CFA, suggesting the presence of an underlying structure in the data. The Chi-Square value of 7025.987 with 231 degrees of freedom and a significance value (Sig.) close to zero (Bartlett's Test) clearly indicate that the correlation matrix is not an identity matrix. This supports the suitability of conducting CFA, as it demonstrates the presence of significant correlations among variables, justifying the repeated application of this method.

Regarding communalities, it is observed that the factor extraction has explained a substantial amount of variance in the observed variables. In general, communalities are moderately high, indicating that the underlying factors in the model are adequately related to the observed variables. This supports the overall validity of the model. It is important to note that some variables, such as D1.2, D2.4, D3.4, and D4.5, have higher communalities, suggesting a strong relationship with the underlying factors. This implies that these variables are particularly relevant for measuring DDC in the context of COMDID A.

Although factor extraction seems appropriate for explaining the variance in the observed variables, it is always essential to consider the validity of the model. If necessary, the possibility of adjusting the number of factors or considering additional factors to improve model fit could be evaluated. For example, some variables, like D3.2 and D4.3, exhibit relatively low communalities, suggesting that they may not be strongly related to the underlying factors and may require further review in terms of their inclusion in the model or conceptualization. Likewise, some variables have loadings on multiple components, indicating they may be related to more than one dimension in the assessment, as observed in the case of variable D1.6.

For future studies, it is recommended to conduct path analysis, structural equation modelling (SEM), or factorial invariance analysis for the selected sample. This would aim to provide a deeper understanding of how the dimensions and constructs of COMDID A relate to and differ among different populations or across different time points.

## Consent

Written informed consent for publication of the participants’ details was obtained from the participants.

## Data Availability

Zenodo: Underlying data for ‘Multivariate data analysis: Validation of an instrument for the evaluation of teaching digital competence. https://doi.org/10.5281/zenodo.10055380 ( Cisneros-Barahona *et al.*, 2023b). The project contains the following underlying data:
• Data File 1: spss data.sav • Data File 2: excel data.xlsx • Data File 3: data project factorial.xlsm (data from the principal component extraction method.) • Data File 4: data project reliability.xlsm (data showing the reliability of the instrument.) •
Figure 1. jpeg •
Figure 2. jpeg •
Figure 3. jpeg •
Figure 4. jpeg Data are available under the terms of the Creative Commons Attribution 4.0 International license (CC-BY 4.0).

## References

[ref1] Almås AG Krumsvik R : Digitally literate teachers in leading edge schools in Norway. *Journal of In-Service Education.* 2007;33(4):479–497. 10.1080/13674580701687864

[ref2] Amaya Amaya A Salazar Blanco M Zúñiga Mireles E : Empoderar a los profesores en su quehacer académico a través de certificaciones internacionales en competencias digitales. *Apertura.* 2018;10(1):104–115. 10.18381/Ap.v10n1.1174

[ref3] Angulo J García RI Torres CA : Nivel de Logro de Competencias Tecnológicas del Profesorado Universitario. *Int. Multiling. J. Contemp. Res.* 2015;3(1):67–80. 10.15640/imjcr.v3n1a8

[ref4] Arias F : *EL PROYECTO DE INVESTIGACIÓN: Guía para su elaboración* (Episteme, Issue January 1997). 1999.

[ref5] Arias F : *EL PROYECTO DE INVESTIGACIÓN: Introducción a la metodología científica* (E. Episteme, Ed.; Sexta Edic, Issue July 2012). 2012.

[ref6] Arnal J Del Rincón D Latorre A : *Investigación Educativa: Fundamentos y metodologías* (Labor S.A.). 1992.

[ref7] Badii MH Castillo J Guillen A : Tamaño óptimo de la muestra Tamaño óptimo de la muestra (Optimum sample size). *InnOvaciOnes de NegOciOs.* 2008;5(1):53–65.

[ref8] Basantes-Andrade A Cabezas-González M Casillas-Martín S : Digital competences relationship between gender and generation of university professors. *International Journal on Advanced Science, Engineering and Information Technology.* 2020;10(1):205–211. 10.18517/ijaseit.10.1.10806

[ref9] Beetham H McGill L Littlejohn A : Thriving in the 21st century: the report of the LLiDA project (Learning Literacies for the Digital Age): Competency frameworks A JISC funded study. June. 2009;1–24.

[ref10] Biel LA Ramos EÁ : Digital teaching competence of the university professor 3.0. *Caracteres.* 2019;8(2):205–236. Reference Source

[ref11] Bisquerra R : Métodos de investigación educativa: Guía práctica. 1989;55–69.

[ref12] Bisquerra R Alzina B Tejedor J Alonso G : Metodología de la Investigación Educativa. *Metodología de la Investigación Educativa (La Muralla).* 2009.

[ref13] Butcher N : Marco de competencias docentes en materia de TIC UNESCO. 2019. Reference Source

[ref14] Cabero-Almenara J Barroso-Osuna J Gutiérrez-Castillo J-J : The Teaching Digital Competence of Health Sciences Teachers. A Study at Andalusian Universities (Spain). *Int. J. Environ. Res. Public Health.* 2021;18(5):2552. 10.3390/ijerph18052552 33806483 PMC7967502

[ref15] Cabero-Almenara J Barroso-Osuna J Rodriguez-Gallego M : Digital Competence for Educators. The case of Andalusian universities. *Aula Abierta.* 2020;49(4):363–372. 10.17811/rifie.49.3.2020.363-372

[ref16] Caena F Redecker C : Aligning teacher competence frameworks to 21st century challenges: The case for the European Digital Competence Framework for Educators (Digcompedu). *Eur. J. Educ.* 2019;54(3):356–369. 10.1111/ejed.12345

[ref17] Campo F Segovia R Martínez P : Competencias TIC para el desarrollo profesional docente. *Ministerio de Educación del Gobierno de Colombia.* 2013.

[ref18] Campo-Arias A : Usos del coeficiente de alfa de Cronbach. *Biomédica.* 2006;26(4):585. 10.7705/biomedica.v26i4.327 17315484

[ref19] Cattell RB : The Scree Test For The Number Of Factors. *Multivar. Behav. Res.* 1966;1(2):245–276. 10.1207/s15327906mbr0102_10 26828106

[ref20] CDEST: Raising the standards: a proposal for the development of an ICT competency framework for teachers. 2002. Reference Source

[ref21] Cervantes V : Interpretaciones del coeficiente Alpha de Cronbach. *Avances En Medición.* 2005;3(January 2005):9–28.

[ref22] Cisneros-Barahona A Marqués Molías L Samaniego-Erazo N : Digital competence, faculty and higher education: Bibliometrics from the Web of Science. *Human Review. International Humanities Review/Revista Internacional de Humanidades.* 2023a, February 8;16(5). 10.37467/revhuman.v12.4680

[ref23] Cisneros-Barahona A Marqués-Molías L Samaniego-Erazo G : Bibliometric Mapping of Scientific Literature Located in Scopus on Teaching Digital Competence in Higher Education. O. S. and R. M. R. and D. C. A. and L.-E. W. Botto-Tobar Miguel and Gómez , editor. *Trends in Artificial Intelligence and Computer Engineering.* Springer Nature Switzerland;2023d; (pp.167–180).

[ref24] Cisneros-Barahona LMM Erazo NIS Granizo CM : Data availability. Multivariate data analysis. Validation of an instrument for the evaluation of teaching digital competence. (Version 3).[Data set]. *Zenodo.* 2023b. 10.5281/zenodo.10055380 PMC10997985 38585228

[ref25] Cisneros-Barahona A Marqués-Molías L Samaniego-Erazo G : Teaching Digital Competence in Higher Education. A Comprehensive Scientific Mapping Analysis with Rstudio. *Commun. Comput. Inf. Sci.* 2022a;14–31. 10.1007/978-3-031-18347-8_2

[ref26] Cisneros-Barahona A Marqués-Molías L Samaniego-Erazo G : Teaching Digital Competences in University Professors: A Meta-analysis and Systematic Literature Review in Web of Science. M. and M. L. S. and T.-C. P. and D. B. Botto-Tobar Miguel and Zambrano Vizuete , editor. *Applied Technologies.* Springer Nature Switzerland;2023; (pp.61–74). 10.1007/978-3-031-24985-3_5

[ref27] Cisneros-Barahona A Marques-Molías L Samaniego-Erazo N : *Evaluación del desempeño del profesor: Propuesta de un Modelo de Rúbrica con base al Sistema de Educación Superior en el Ecuador.* Thomson Reuters;2022.

[ref28] Cisneros-Barahona A Marqués-Molías L Samaniego-Erazo N : Digital competence of university teachers. An overview of the state of the art. *HUMAN REVIEW. International Humanities Review/Revista Internacional de Humanidades.* 2022b, December 27;11(Monográfico):1–25. 10.37467/revhuman.v11.4355

[ref29] Cronbach LJ : Coefficient alpha and the internal structure of tests. *Psychometrika.* Springer-Verlag;1951; (Vol.16(3). 10.1007/BF02310555

[ref30] De la Iglesia JCF Fernández-Morante MC Cebreiro B : Competences and attitudes for the use of ICT in Galician students of the degree of teaching. *Publicaciones de La Facultad de Educacion y Humanidades Del Campus de Melilla.* 2020;50(1):103–120. 10.30827/PUBLICACIONES.V50I1.11526

[ref31] Delfín M Pirela G : Software Tool for Bibliometric and Network Analyses of Scientific Production. *Códices.* 2017;13(1):109–125. Reference Source

[ref32] Domingo-Coscollola M Bosco A Segovia SC : Fostering teacher’s digital competence at university: The perception of students and teachers. *Revista de Investigacion Educativa.* 2020;38(1):167–182. 10.6018/rie.340551

[ref33] Elliot J Gorichon S Irigoin M : Competencias y Estándares TIC para la Profesión Docente. 2011. Reference Source

[ref34] Fernández Cruz F Fernández Díaz M : Los docentes de la Generación Z y sus competencias digitales. *Comunicar: Revista Científica Iberoamericana de Comunicación y Educación.* 2016;46:97–105.

[ref35] Fernández-Diaz M Robles-Moral FJ Ayuso-Fernández GE : Una propuesta para trabajar la competencia digital docente a través de Instagram y el Pensamiento Visual: el estudio de la sostenibilidad. *Revista Latinoamericana de Tecnología Educativa - RELATEC.* 2021;20(1):87–102. 10.17398/1695-288X.20.1.87

[ref36] Fernández-Márquez E Leiva-Olivencia J Lopez-Meneses E : Digital Competences in Higher Education Professors. *REVISTA DIGITAL DE INVESTIGACION EN DOCENCIA UNIVERSITARIA-RIDU.* 2018;12(1):213–231. 10.19083/ridu.12.558

[ref37] Ferrari A : Digital Competence in Practice: An Analysis of Frameworks. *Joint Research Centre of the European Commission.* 2013;91. 10.2791/82116

[ref38] Galindo-Domínguez H Bezanilla MJ : Digital competence in the training of pre-service teachers: Perceptions of students in the degrees of early childhood education and primary education. *J. Digit. Learn. Teach. Educ.* 2021;37:262–278. 10.1080/21532974.2021.1934757

[ref39] García-Ruiz R Buenestado-Fernández M Ramírez-Montoya MS : Evaluación de la Competencia Digital Docente: instrumentos, resultados y propuestas. Revisión sistemática de la literatura. *Educación XX1.* 2023;26(1):273–301. 10.5944/educxx1.33520

[ref40] Garita-González G Gutierrez-Durán J-E Godoy-Sandoval V : Teaching perception on digital competencies and pedagogical mediation applied the elaboration of didactic material of the Cátedra de Administración de la Universidad Estatal a Distancia (UNED). *Revista Electrónica Calidad En La Educación Superior.* 2019;10(1):125–159. 10.22458/caes.v10i1.2181

[ref41] Garmendía M : Análisis factorial: una aplicación en el cuestionario de salud general de Goldberg, versión de 12 preguntas*. *Rev. Chil. Salud Pública.* 2007;11(2):57–65.

[ref86] Gisbert Cervera M Esteve Mon F : Digital Leaners: la competencia digital de los estudiantes universitarios. *La Cuestión Universitaria.* 2011;7(May):48–59. https://bit.ly/45XaJke

[ref42] Gisbert Cervera M González Martínez J Esteve Mon FM : Competencia digital y competencia digital docente: una panorámica sobre el estado de la cuestión. *Revista Interuniversitaria de Investigación En Tecnología Educativa.* 2016;74–83. 10.6018/riite2016/257631

[ref43] González J Pazmiño M : Cálculo e interpretación del Alfa de Cronbach para el caso de validación de la consistencia interna de un cuestionario, con dos posibles escalas tipo Likert. *Revista Publicando.* 2015. Reference Source

[ref44] Guillén-Gámez F Mayorga-Fernández M Contreras-Rosado J : Incidence of gender in the digital competence of higher education teachers in research work: Analysis with descriptive and comparative methods. *Education Sciences.* 2021;11(3):1–14. 10.3390/educsci11030098

[ref85] Gutiérrez I : Competencias del profesorado universitario en relación al uso de tecnologías de la información y comunicación: análisis de la situación en España y propuesta de un modelo de formación. In [Tesis Doctoral] Universitat Rovira I Virgili. Departamento de Pedagogía;2011.

[ref45] Gutiérrez J Cabero-Almenara J : A Case study self-percepcion digital competence of the university student in Bachelor’s degrees in the Pre-School Teacher Education and Primary. 2016;2.

[ref46] Hair JR Black JF Babin WC : Multivariate Data Analysis. 2010.

[ref47] Hernández Sampieri R Fernández Collado C Baptista Lucio P : Capítulo 12. Ampliación y fundamentación de los métodos mixtos. 2014a.

[ref48] Hernández Sampieri R Fernández Collado C Baptista Lucio P : *Metodología de la investigación.* McGRAW-H;2014b.

[ref49] INTEF: Marco Común de Competencia Digital Docente. 2017. Reference Source

[ref50] ISTE: The ISTE National Educational Technology Standards (NETS•S) and Performance Indicators for Students Essential Conditions Necessary conditions to effectively leverage technology for learning Shared Vision. 2000. Reference Source

[ref51] ISTE: Crosswalk: Future Ready Librarians Framework and ISTE Standards for Educators. September. 2008.

[ref52] Juárez Arall J Marqués Molías L : Aspectos de la competencia digital para la empleabilidad//Digital competence aspects for employability. *REOP - Revista Española de Orientación y Psicopedagogía.* 2019;30(2):67. 10.5944/reop.vol.30.num.2.2019.25339

[ref53] Kerlinger F Lee H : *Investigación del comportamiento.* McGRAW-HIL;1985.

[ref54] Larraz V : *La competència digital a la Universitat.* Universidad de Andorra;2013. Reference Source

[ref55] Lázaro J Gisbert M Silva J : Una rúbrica para evaluar la competencia digital del profesor universitario en el contexto latinoamericano. *Edutec. Revista Electrónica de Tecnología Educativa.* 2018;63: 10.21556/edutec.2018.63.1091

[ref56] Lázaro L Gisbert M : Elaboración de una rúbrica para evaluar la competencia digital del docente. *UT. Revista de Ciències de l’Educació.* 2015. Reference Source

[ref58] Lázaro-Cantabrana JL Gisbert-Cervera M Silva-Quiroz JE : Una rúbrica para evaluar la competencia digital del profesor universitario en el contexto latinoamericano. *Edutec. Revista Electrónica de Tecnología Educativa.* 2018;63. 10.21556/edutec.2018.63.1091

[ref59] Lázaro-Cantabrana JL Usart M Cervera MG : Assessing teacher digital competence: The construction of an instrument for measuring the knowledge of pre-service teachers. *Journal of New Approaches in Educational Research.* 2019;8(1):73–78. 10.7821/naer.2019.1.370

[ref60] Ledesma R Molina G Valero P : Análisis de consistencia interna mediante Alfa de Cronbach: un programa basado en gráficos dinámicos. *Psico-USF.* 2002;7:143–152. 10.1590/S1413-82712002000200003

[ref61] López-Aguado M Gutiérrez-Provecho L : Cómo realizar e interpretar un análisis factorial exploratorio utilizando SPSS. *REIRE Revista d Innovaciói Recerca En Educació.* 2019;12(2). 10.1344/reire2019.12.227057

[ref62] Malagón Terrón FJ Graell Martín M : La formación continua del profesorado en los planes estratégicos de las universidades españolas. *Educación XX1.* 2022;25(1):433–458. 10.5944/educxx1.30321

[ref63] Marqués-Molías L Esteve-González V Holgado-Garcia J : Student perceptions of ePortfolio as competence assessment during the practical training period for early childhood and primary school teaching. *Proceedings of the European Conference on E-Learning, ECEL*, *2016-Janua.* 2016; (1):777–781.

[ref64] Mateos-Aparicio G Hernández Estrada A : Análisis multivariante de datos. Cómo buscar patrones de comportamiento en BIG DATA. 2021.

[ref65] Merino Soto C Lautenschlager G : Comparación Estadística de la Confiabilidad Alfa de Cronbach: Aplicaciones en la Medición Educacional y Psicológica. *Revista de Psicología.* 2003;12(2):127. 10.5354/0719-0581.2003.17668

[ref66] Morales Capilla M Trujillo Torres JM Raso Sánchez F : Percepciones acerca de la integración de las TIC en el proceso de enseñanza-aprendizaje de la universidad. *Píxel-Bit, Revista de Medios y Educación.* 2014;46:103–117. 10.12795/pixelbit.2015.i46.07

[ref67] Palau R Usart M Ucar Carnicero MJ : The digital competence of teachers in music conservatories. A study of self-perception in Spain. *Revista Electronica de LEEME.* 2019;44:24–41. 10.7203/LEEME.44.15709

[ref68] Pérez ER Medrano L : Análisis Factorial Exploratorio: Bases Conceptuales y Metodológicas Artículo de Revisión. *Revista Argentina de Ciencias Del Comportamiento.* 2010;2:58–66. Reference Source

[ref69] Ramírez García A González Fernández N : Competencia mediática del profesorado y del alumnado de educación obligatoria en España. *Comunicar: Revista Científica Iberoamericana de Comunicación y Educación.* 2016;49:49–58.

[ref70] Ramos-Galarza CA : Alcances de una investigación. *CienciAmérica.* 2020;9(3):1–6. 10.33210/ca.v9i3.336

[ref71] Rangel A : Propuesta De Un Perfil Digital Teaching Skills: a Profile. *Pixel-Bit. Revista de Medios y Educación.* 2015;235–248.

[ref72] Redecker C : European framework for the digital competence of educators: DigCompEdu. *Joint Research Centre (JRC) Science for Policy report.* 2017. 10.2760/159770

[ref73] Reyes J Cárdenas M Díaz Ocampo E : Las Competencias Digitales: una necesidad del docente Ecuatoriano del siglo XXI. *Evista Dilemas Contemporáneos: Educación, Política y Valores.* 2018;12–26. Reference Source

[ref74] Sánchez-Caballé A Gisbert-Cervera M Esteve-Mon F : The digital competence of university students: a systematic literature review. *Aloma: Revista de Psicologia, Ciències de l’Educació i de l’Esport.* 2020;38(1):63–74. 10.51698/aloma.2020.38.1.63-74

[ref75] Silva J Usart M Lázaro-Cantabrana J-L : Teacher’s digital competence among final year Pedagogy students in Chile and Uruguay. *Comunicar.* 2019;27(61):33–43. 10.3916/C61-2019-03

[ref76] Silva Quiroz J Miranda Arredondo P : Presencia de la competencia digital docente en los programas de formación inicial en universidades públicas chilenas. *Revista de Estudios y Experiencias En Educación.* 2020;19(41):149–165. 10.21703/rexe.20201941silva9

[ref77] Torres J : Fiabilidad de las escalas: interpretación y limitaciones del Alfa de Cronbach. 2021. Reference Source

[ref78] Trilling B : 21st CENTURY STUDENT OUTCOMES. 2002. Reference Source

[ref80] Usart Rodríguez M Lázaro Cantabrana JL Gisbert Cervera M : Validation of a tool for self-evaluating teacher digital competence. *Educación XX1.* 2020;24(1):353–373. 10.5944/educxx1.27080

[ref81] Verdú-Pina M Usart M Grimalt-Álvaro C : Report on the process for evaluating and certifying Teacher Digital Competence An international perspective. 2022. Reference Source

[ref82] Vivar DM : Ramón Cózar Gutiérrez y M ^a^ del Valle de Moya Martínez (coords.) (2013) Las TIC en el aula desde un enfoque multidisciplinar. Aplicaciones practices. Barcelona: Octaedro. *ENSAYOS. Revista de La Facultad de Educación de Albacete.* 2014;29(2):181–182. 10.18239/ensayos.v29i2.645

[ref83] Zepeda H Méndez ME Galván H : Evaluación de la Competencia Digital en Profesores de Educación Superior de la Costa Norte de Jalisco. *Revista Iberoamericana de Producción Académica y Gestión Educativa.* 2019;6(11).

[ref84] Zhao Y Pinto Llorente AM Sánchez Gómez MC : The impact of gender and years of teaching experience on college teachers’ digital competence: an empirical study on teachers in gansu agricultural university. *Sustainability (Switzerland).* 2021;13(8):4163. 10.3390/su13084163

