# Supplementary material for: Multivariate data analysis: Validation of an instrument for the evaluation of teaching digital competence
Source: F1000Res. 2023 Nov 6;12:866. Originally published 2023 Jul 21. [Version 2] doi: 10.12688/f1000research.135194.2 (PMC10997985; doi:10.12688/f1000research.135194.2)
Supplement: Peer review history

# Reviewer report: Reviewer response for version 2

**DOI:** 10.5256/f1000research.158348.r221088

**Published:** 2023-11-22

**Authors:** Francesc M. Esteve-Mon

**Affiliations:** 1 Jaume I University, Castellón de la Plana, Spain

The current manuscript is adequate and relevant to the scientific field of educational technology. 

 The link between digital competence (DC) and teaching digital competence (TDC) in the theoretical framework has been improved. The method and purpose have been restructured and the information of the instrument has been correctly expanded. Likewise, the conclusions and implications have been satisfactorily expanded.

Is the work clearly and accurately presented and does it cite the current literature?

Partly

If applicable, is the statistical analysis and its interpretation appropriate?

Yes

Are all the source data underlying the results available to ensure full reproducibility?

Yes

Is the study design appropriate and is the work technically sound?

Yes

Are the conclusions drawn adequately supported by the results?

Partly

Are sufficient details of methods and analysis provided to allow replication by others?

Yes

Reviewer Expertise:

Educational technology, digital competence and teacher training.

I confirm that I have read this submission and believe that I have an appropriate level of expertise to confirm that it is of an acceptable scientific standard.

# Response

**Published:** 2023-11-29

**Authors:** Andres Santiago Cisneros Barahona

**Affiliations:** Ingeniería, Universidad Nacional de Chimborazo, Riobamba, Chimborazo Province, Ecuador

Thank you for your valuable contributions, which have enriched the manuscript.

# Reviewer report: Reviewer response for version 1

**DOI:** 10.5256/f1000research.148298.r191135

**Published:** 2024-04-05

**Authors:** Amaia Arroyo Sagasta

**Affiliations:** 1 Mondragon University, Mondragon, Spain

This article explains the validation process of the COMDID A instrument for the assessment of Teaching Digital Competence (TDC). It describes deeply the method used for reaching that validation, based on a descriptive-correlational scope and a non-experimental-cross-sectional design. The main conclusion highlights that the reliability is confirmed, also the dimensionality. 

 As a contribution to the article, I would add some more information in the introduction and conclusion sections. In the introduction, some more information about the COMDID framework would be appreciated for those researches who don't know much about it. In the case of the conclusion section, the last key idea can be developed and some other research lines for the future can be cited.

Is the work clearly and accurately presented and does it cite the current literature?

Yes

If applicable, is the statistical analysis and its interpretation appropriate?

I cannot comment. A qualified statistician is required.

Are all the source data underlying the results available to ensure full reproducibility?

Yes

Is the study design appropriate and is the work technically sound?

Yes

Are the conclusions drawn adequately supported by the results?

Partly

Are sufficient details of methods and analysis provided to allow replication by others?

Yes

Reviewer Expertise:

Education, Digital Competence, Teacher Digital Competence, Technopedagogy

I confirm that I have read this submission and believe that I have an appropriate level of expertise to confirm that it is of an acceptable scientific standard.

# Reviewer report: Reviewer response for version 1

**DOI:** 10.5256/f1000research.148298.r191131

**Published:** 2023-09-22

**Authors:** Francesc M. Esteve-Mon

**Affiliations:** 1 Jaume I University, Castellón de la Plana, Spain

This paper discusses a relevant and important topic on the evaluation of teaching digital competence. It is well written and in general very clear.

 Still I have comments:

- The authors provide a well-founded conceptual and state-of-the-art overview. However, there are some references that should be revised in the Introduction section. There are some citations that, although they may be related to the research, are a bit forced. These citations are not directly related to the authors' argument and should be reviewed. Furthermore, in the theoretical framework (Introduction), the link between digital competence (DC) and teaching digital competence (TDC) should be reviewed. 
- Also, the purpose of the article should be made clearer as the final part of the Introduction. Right now the aim is in the Ethical Considerations section of the Method. It should be restructured. In addition, the details of the instrument should be in the Method section. 
- Beyond the analysis and discussion of statistical results, it is important to broaden the discussion in terms of progress and impact in the field of TDC. Overall, the manuscript makes some interesting points, but conclusions and implications could be extended.

Minor comments:

- Please review the acronyms used and define them at the first mention. For example, digital competence (DC).

Is the work clearly and accurately presented and does it cite the current literature?

Partly

If applicable, is the statistical analysis and its interpretation appropriate?

Yes

Are all the source data underlying the results available to ensure full reproducibility?

Yes

Is the study design appropriate and is the work technically sound?

Yes

Are the conclusions drawn adequately supported by the results?

Partly

Are sufficient details of methods and analysis provided to allow replication by others?

Yes

Reviewer Expertise:

Educational technology, digital competence and teacher training.

I confirm that I have read this submission and believe that I have an appropriate level of expertise to confirm that it is of an acceptable scientific standard, however I have significant reservations, as outlined above.

# Response

**Published:** 2023-10-30

**Authors:** Andres Santiago Cisneros Barahona

**Affiliations:** Ingeniería, Universidad Nacional de Chimborazo, Riobamba, Chimborazo Province, Ecuador

- The authors provide a well-founded conceptual and state-of-the-art overview. However, there are some references that should be revised in the Introduction section. There are some citations that, although they may be related to the research, are a bit forced. These citations are not directly related to the authors' argument and should be reviewed. Furthermore, in the theoretical framework (Introduction), the link between digital competence (DC) and teaching digital competence (TDC) should be reviewed. 

Definitions linking TDC and DC have been added to the Introduction section.

- Also, the purpose of the article should be made clearer as the final part of the Introduction. Right now the aim is in the Ethical Considerations section of the Method. It should be restructured. In addition, the details of the instrument should be in the Method section. 

The research objective has been added to the end of the Introduction section. Additionally, in the Methods section, details regarding the chosen instrument have been included, along with the justification for its selection.

- Beyond the analysis and discussion of statistical results, it is important to broaden the discussion in terms of progress and impact in the field of TDC. Overall, the manuscript makes some interesting points, but conclusions and implications could be extended.

The Results and Conclusions sections have been expanded to highlight the relevance of the study in relation to TDC.

 Minor comments:

- Please review the acronyms used and define them at the first mention. For example, digital competence (DC).

The acronyms for Digital Competence (DC) and Teacher Digital Competence (TDC) are defined in the abstract of the article

# Reviewer report: Reviewer response for version 1

**DOI:** 10.5256/f1000research.148298.r200607

**Published:** 2023-09-12

**Authors:** Yu Zhao

**Affiliations:** 1 Universidad de Salamanca, Salamanca, Castile and León, Spain

This paper focused on the validation of an instrument for the evaluation of teaching digital competence. It is an interesting topic and the article is easy to read.

 However, there are some issues that need further improvement and revision:

1. What's the difference between digital competence and teaching digital competence? Since COMDID focuses mainly on teaching digital competence, it should be mentioned in the introduction part.
2. The COMDID tool has been mentioned and used in several works, why did the authors choose this tool for validation?
3. The objective of this study is to validate the COMDID to assess TDC, but  the findings do not seem to respond well to the purpose of the study.
4. The limitations of the study need to be mentioned.
5. The manuscript requires thorough proofreading.

Is the work clearly and accurately presented and does it cite the current literature?

Partly

If applicable, is the statistical analysis and its interpretation appropriate?

I cannot comment. A qualified statistician is required.

Are all the source data underlying the results available to ensure full reproducibility?

Yes

Is the study design appropriate and is the work technically sound?

Yes

Are the conclusions drawn adequately supported by the results?

Partly

Are sufficient details of methods and analysis provided to allow replication by others?

Partly

Reviewer Expertise:

ICT, digital competence, language learning and teaching.

I confirm that I have read this submission and believe that I have an appropriate level of expertise to confirm that it is of an acceptable scientific standard, however I have significant reservations, as outlined above.

# Response

**Published:** 2023-10-30

**Authors:** Andres Santiago Cisneros Barahona

**Affiliations:** Ingeniería, Universidad Nacional de Chimborazo, Riobamba, Chimborazo Province, Ecuador

- What's the difference between digital competence and teaching digital competence? Since COMDID focuses mainly on teaching digital competence, it should be mentioned in the introduction part.

In the introduction section, the difference between the definition of DC and TDC has been added.

- The COMDID tool has been mentioned and used in several works, why did the authors choose this tool for validation?

In the Methods section, the justification for the choice of the instrument has been added 

- The objective of this study is to validate the COMDID to assess TDC, but  the findings do not seem to respond well to the purpose of the study.

In the Results and Conclusions section, the relevant aspects that fulfill the research objective have been added.

- The limitations of the study need to be mentioned.

The limitations of the study have been added in the Methods section. 

- The manuscript requires thorough proofreading.

The manuscript has undergone a comprehensive review.
